# Seasonal distribution and seven year trend of malaria in North West Tigrai: 2012–2018, Ethiopia; 2019

**DOI:** 10.1186/s40794-019-0091-y

**Published:** 2019-08-14

**Authors:** Brhane Berhe, Fitsum Mardu, Haftom Legese, Hadush Negash

**Affiliations:** Department of Medical Laboratory Sciences, College of Medicine and Health Science, Adigrat University, Adigrat, Ethiopia

**Keywords:** Ethiopia, Malaria trend, Meteorological data, Seasonal distribution, Suhul, Tigrai

## Abstract

**Background:**

Malaria is a serious public concern in Ethiopia, 75% of the land and 60% of the population are exposed to the disease. The disease has been consistently reported as one of the top three leading causes of outpatient visits, admissions, and deaths among all age group in Ethiopia. However, there is no published data to date regarding the trends of malaria in north western Tigrai, northern Ethiopia. Hence, knowing the trends of malaria prevalence in this area is essential to design appropriate interventions against the disease.

**Methods:**

Institutional based retrospective study was conducted to determine trends in prevalence of malaria from documented laboratory logbooks at Suhul General Hospital, northwestern Tigrai, northern Ethiopia. All recorded malaria cases from January 2012 to December 2018 were carefully reviewed and analyzed from the laboratory logbooks. Additionally, any malaria intervention activities applied in the area were collected by a checklist. Beside, data related to temperature and rainfalls were collected from metrological office of Shire-endasilasie town.

**Results:**

During the seven years (2012–2018) data, a total of 71,986 blood films were requested for malaria diagnosis in Suhul Hospital and 5010(6.96%) microscopically confirmed malaria cases reported in the study area with fluctuating trends. *Plasmodium falciparum* and *Plasmodium vivax* were the dominant parasites detected, which accounted (2516; 50.2%, 2181; 43.5%) respectively. However, individuals aged ≥15 years (3628; 72.4%) and male participants (3142, 62.7%) were found highly infected with malaria parasites. Despite the yearly abundance of malaria cases, highest prevalence was reported in autumn (September–November) in the study area.

**Conclusions:**

Malaria is still a major health dilemma Northwestern Tigrai, Northern Ethiopia. *Plasmodium falciparum* and *Plasmodium vivax* were unmoving predominant parasite reported in the study area. Overall, trend of malaria over the years showed no significant reduction or increment. So, strong scaling up of the community should going on towards transmission, prevention and control activities of malaria in view of *Plasmodium falciparum* and *Plasmodium vivax.*

## Background

Malaria is the most serious public health concern in developing countries. World Health Organization report showed that half of the world’s population and almost all of the African populations are at risk of malaria [1–3]. The dilemma of malaria is very severe in Ethiopia where it had been the major cause of morbidity and mortality for many years [4]. It leads more than 50 million people at risk [5], and four to five million people are affected annually in the country [6].

In Ethiopia, malaria is a leading alarm and has been consistently reported as one of the three leading causes of morbidity and mortality. It was also a leading cause of outpatient visits, admissions, and deaths in the country from 2005 to 2006 accounting for over 20% of the deaths at all ages [7]. There are about 5–6 million annual confirmed malaria cases in Ethiopia; *Plasmodium falciparum* and *Plasmodium vivax* being the most common species identified [8].

About 75% of the land and 60% of the population is exposed to malaria in Ethiopia. There is low-to-moderate malaria transmission in the country. However, due to the unstable and seasonal transmission of malaria in the country, protective immunity of the population is generally low and all age groups are at risk with an estimated prevalence of 1.8% [6].

The Federal Ministry of Health of Ethiopia (FMOH) estimated that there are about 12 million suspected for malaria in the country each year. A total of 3,384,589 malaria cases (from 2011 to 2012) were reported with 1,793,832(53%) and 1,590,757(47%) of them were laboratory and clinically confirmed cases respectively. *Plasmodium falciparum* (1,061,242(59.2%) and *Plasmodium vivax* (732,590 (40.8%) were the only species reported. There were also 936 malaria deaths reported in the country in 2012 [9].

According to Health Management Information System (HMIS) data of Tigrai region, the total Out Patient Department (OPD) visits, admissions and deaths due to malaria was decreased from 20.5, 10.5 and 5.1% in 2011/2012 to 11.6, 4.4 and 1.9% in 2014/15. On the contrary, the incidence of malaria in Tigrai region is about 8.1 from 100,000 at risk population with outbreaks occurred in Humera, Asgede Tsimbila and Tahtay adiyabo areas [10].

Despite, Ethiopia has achieved remarkable progress in the fight against malaria during the most recent decades through case management interventions. This was achieved by the involvement of Health Extension Workers (HEWs) and Health Development Army (HAD) volunteers providing community based care at the household level [11]. In children under five years of age, malaria admissions and deaths fell by 81% in 2001 to 73% in 2011. However, malaria still remains a serious headache in Ethiopia due to different factors such as sub-optimal uptake of anti-malarial intervention, poor data quality and utilization, quality of indoor residual spray operation and environmental compliance, vector insecticide resistance, low coverage of malaria impeding service, poor access to health care, increase trend in internal displacement of people and migrant workers, refuge influx, and limited financial and human resources [12–14]. Therefore, this study is aimed to assess the prevalence and trend of malaria in northwestern Tigrai, Ethiopia from 2012 to 2018. Beside, this study will gives scientific evidence for local, national and universal in advancing current knowledge on malaria situation in Ethiopia. It is also useful for policy makers and program planners in preventing and controlling malaria cases.

## Methods

### Study design, period and area

An institutional based retrospective cross-sectional study was conducted in Suhul General Hospital, northwest Tigrai, from January 2012 to December 2018. The laboratory logbooks of malaria were reviewed to determine a seven-year trend in prevalence of malaria in the study area. Suhul General Hospital is found in Shire-endasilasie town, located around 1071 km north of Addis Ababa (capital of the country) at a latitude and longitude of 14°6′N 38°17′E, at an elevation of 1953 m (4017 ft) above sea level. It has a tropical savanna climate with average annual rainfall reaching 905 mm. The average maximum and minimum temperature of the town is 28.2^o^c and 12.5^o^c respectively. Based on the 2007 Census conducted by the Central Statistical Agency of Ethiopia (CSA), Tigrai has a total population of 5.23 million. Shire-endasilasie has a total population of 47,284, of whom 25,417 are women. The economic status of Tigrai region depends mainly on agricultural activities (farming and gold mining). In the region there are areas for tourists to be visited like obelisks of Aksum, Geralta Mountain, Mountains of Adwa, Rock hewn churches and Atse Yohannes palace. Suhul General Hospital has 34 health facilities as catchment areas. So, Suhul General Hospital is the only General hospital in North-western Tigrai which services for high number of people. Besides, the town is located near to the malaria endemic areas like Humera and Sheraro towns where there is a history of new malaria cases [10]. Currently, the hospital is serving as a teaching hospital, emergency, inpatient and outpatients services for many people.

### Inclusion criteria

Data such as number of malaria cases diagnosed in months and years, types of malaria species identified, and socio-demographic data (age and sex) were included in the analysis regardless of age, pregnancy and other infection status.

#### Exclusion criteria

Any missing data in the inclusion criteria were excluded.

#### Sample size

All of the 71,986 malaria reports registered from January 2012 to December 2018 in the laboratory logbook that fulfilled the inclusion criteria were taken for analysis.

## Data collection

### Malaria data

Seven-year malaria data (from January 2012 to December 2018) were collected from Suhul General Hospital laboratory logbooks. Malaria cases diagnosed in months and years, types of malaria species identified, and socio-demographic data such as age and sex were collected by trained five Medical laboratory technologists.

The laboratory staffs of Suhul General Hospital were using well prepared and staining peripheral microscopy smear examination as a gold standard diagnostic method for the detection and species identification of plasmodium parasites. The hospital strictly follows the standard operating procedures (SOPs) for capillary and venous blood sample collection, smear preparation, staining and blood film examination for malaria parasite detection in all phases of the quality control. In the study area, thin smears are considered positive for malaria if one or more plasmodium parasites are seen; and no hemoparasites if no parasite was observed in 200 high-power fields. On the other hand, thick blood films were taken as positive if one or more plasmodium parasites are observed; and no hemoparasites if no parasites were seen after examining 100 high-power fields. Besides, the seven-year climatic condition such as temperature and rain fall was collected from metrological data office of Shire-endasilasie Wereda, north western Tigrai.

### Malaria intervention taken in the study area

Malaria control and preventive measures taken in each year (from January 2012–December 2018) were assessed using a checklist adopted from different literatures [8, 9].

Some of the interventions taken were;

- ❖ Creating awareness of the community through health education on the application of insecticide treated bed nets and repellents (like buzz off) (2004–2014)
- ❖ Increased accessibility of insecticide treated bed nets to the community (2004–2014)
- ❖ Increment of budget for malaria prevention and control activities by FMOH, Regional Health Bureau and different stakeholders (obtained through personal communication) (2005–2013)
- ❖ Different Public conferences that were arranged by FMOH and regional health bureau were held to address and promote insecticide treated bed nets utilization and environmental management activities (2016–2018)
- ❖ Most case managements of *P. falciparum* and *P. vivax* were carried out with coartem and chloroquine (obtained through personal communication) (2004–2019)
- ❖ Weekly active malaria case surveillance was reported (2010–2019)

### Quality control

To assure the quality of data, we first assessed the completeness of the malaria logbooks in the laboratory. Appropriately designed data collection format sheet was prepared and used for data recording. The data collectors were adequately informed about the data collection process. Each day the data collected, we reviewed and checked for completeness and consistency of the information. Besides, all of the laboratory personnel in the hospital had more than 3 years experience in malaria microscopy; they had also additional training on malaria microscopy at least once in their career and the staff themselves had weekly schedule gap filling seminars on selected topics like malaria laboratory diagnosis and shared their experience.

### Data analysis

Data was entered to Microsoft office excels worksheet 2007 and analyzed using SPSS version 22. Frequency distributions of both dependant and independent variables were calculated using cross tabulation. Finally, the data were summarized and presented in the form of figures and tables.

### Ethical consideration

Ethical clearance was obtained from Research and Community service Directorate of Adigrat University, Adigrat, Ethiopia. After discussing the purpose and relevance of the study, we have got written permission letter from the administration of Suhul General Hospital before staring the data collection.

## Results

### Trend of malaria prevalence in Northwestern Tigrai from 2012 to 2018

**Fig. 1:**
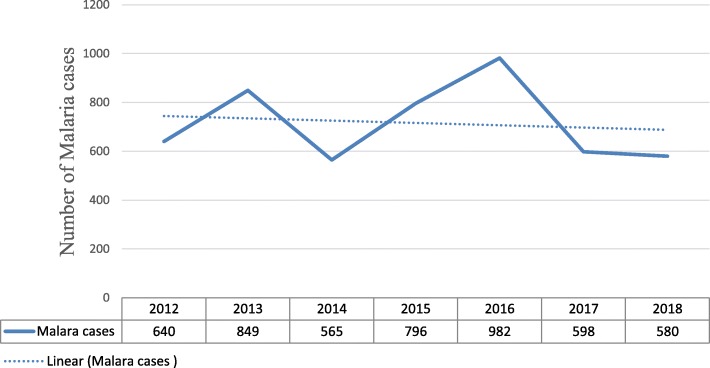
Trend of malaria cases in Suhul General Hospital from 2012 to 2018

During the study period (2012 to 2018), a total of 71,986 blood films from malaria suspected patients were examined in Suhul General Hospital. Of these, 5010 (6.96%) were microscopically confirmed malaria cases with the mean annual malaria case number of 716. The prevalence of malaria was fluctuating during the seven years of study with minimum (*n* = 565) and maximum (*n* = 982) number of annual cases reported by the years 2014 and 2016 respectively (Fig. 1). The highest (*n* = 11,831) and lowest (*n* = 9145) number of malaria-suspected patients were examined in 2016 and, 2015 respectively.

The prevalence had slightly risen from 2012 to 2013 and then dropped off from 2013 to 2014. After that, it sharply increased from 2014 to 2016. Finally, the prevalence had fallen until 2018. Overall, this finding indicated that, no significant reduction in laboratory confirmed malaria cases was recorded from 2012 to 2018 in Northwestern Tigrai, (Fig. 1).

**Fig. 2:**
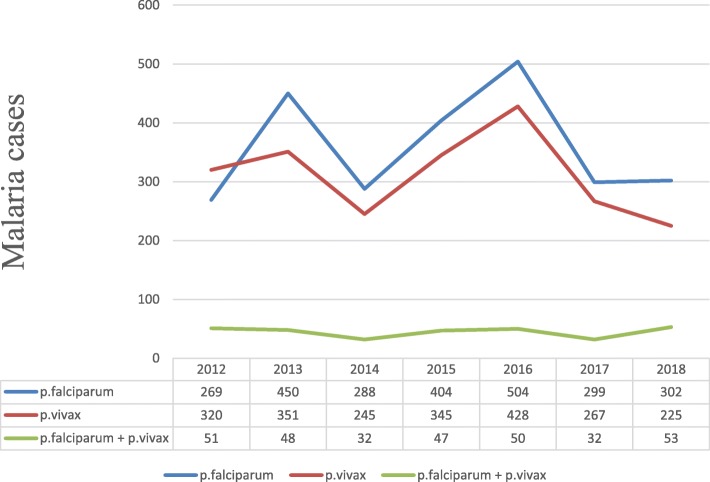
Trend of plasmodium species in Suhul General Hospital from 2012 to 2018

*Plasmodium falciparum* was the most commonly reported species accounted for 2516 (50.2%) of the cases while *Plasmodium vivax* was reported among 2181(43.5%) of the positive results. Mixed (*P.falciparum* and *P.vivax*) infections were reported in 313(6.3%) of the confirmed cases. *Plasmodium falciparum* and *Plasmodium vivax* showed gradual increment from 2014 to 2016 while *Plasmodium falciparum* was reduced by 40% from the year 2016 to 2017 (Fig. 2).

### Prevalence of Plasmodium parasites in relation to age and sex of individuals in northwestern Tigrai

Of the 5010 confirmed malaria cases, 3427(68.4%) were reported among males while 1583 (31.6%) were reported among females with a male to female ratio of 2.02. During the reviewed period, all age groups were affected by malaria with the higher prevalence 3628(72.4%) occurred among individuals aged ≥15 years old, followed by the age group of 5–14 years, 634(12.7%), and < 5 years, 472(9.4%) (Table 1). *Plasmodium falciparum* was commonly reported in most age groups except in the 5–14 years age groups. It dominantly affected individuals aged fifteen years or older (39.6%, 1983/5010). Similarly, high prevalence of *Plasmodium vivax* was also reported among individuals in the age groups≥15 years of age (Table 1).

**Table 1:** Prevalence of plasmodium species in different age categories and sex of patients in Suhul Hospital from 2012 to 2018

Variables	PF n (%)	PV n (%)	Mixed infection (PF + PV) n (%)	Total (%)
Sex				
Male	1600(32)	1542(31)	285(5.7)	3427(68.4)
Female	916(18.2)	639(12.8)	28(0.56)	1583(31.6)
Age category				
< 5	248(5)	187(3.7)	8(0.16)	443(8.8)
5–14	285(5.7)	349(6.97)	17(0.34)	651(12.9)
≥15	1984(39.6)	1644(32.8)	288(5.7)	3916(78.2)

### Seasonal distribution of malaria based on temperature and rainfall in Suhul general hospital

**Fig. 3:**
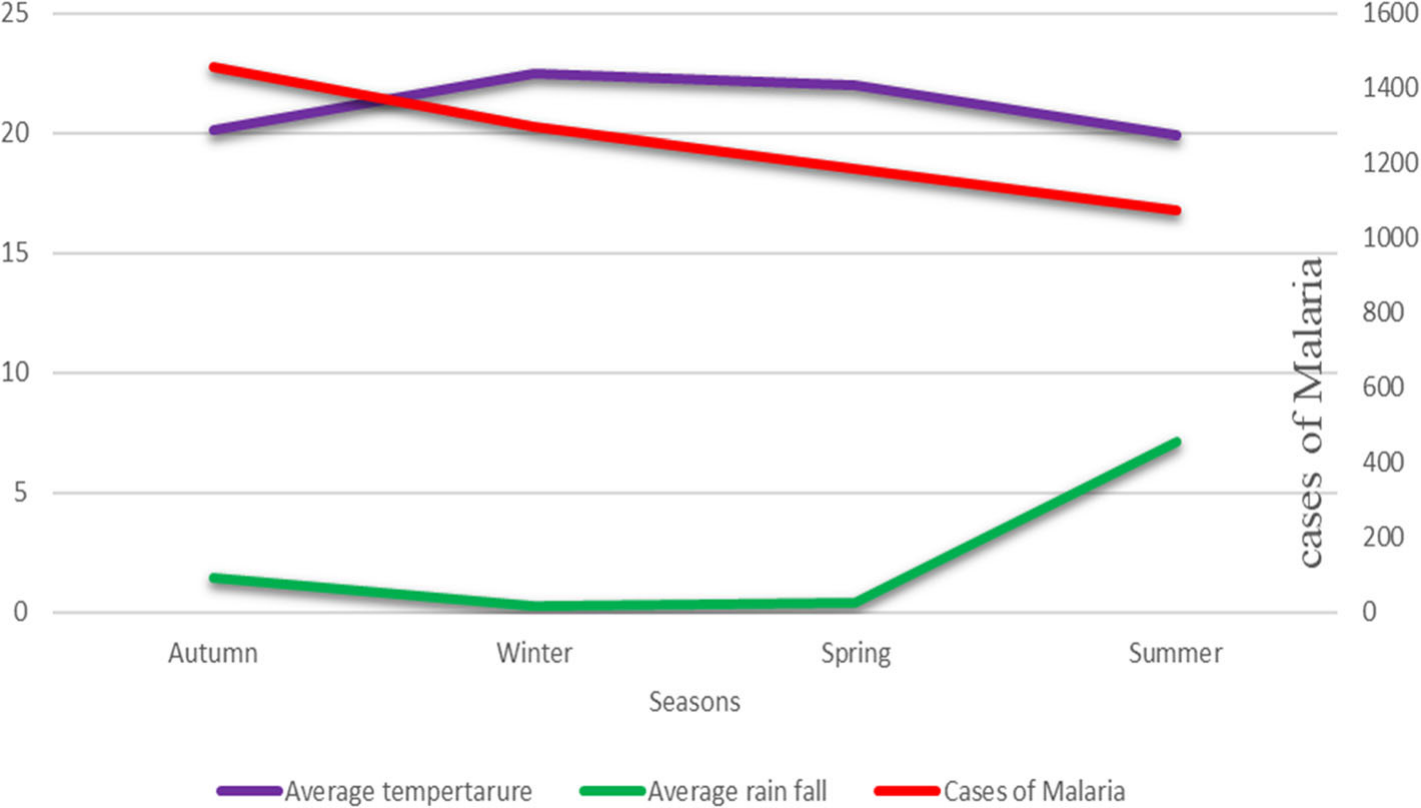
Seasonal distribution of malaria based on rainfall and temperature in Suhul General Hospital from 2012 to 2018

**Fig. 4:**
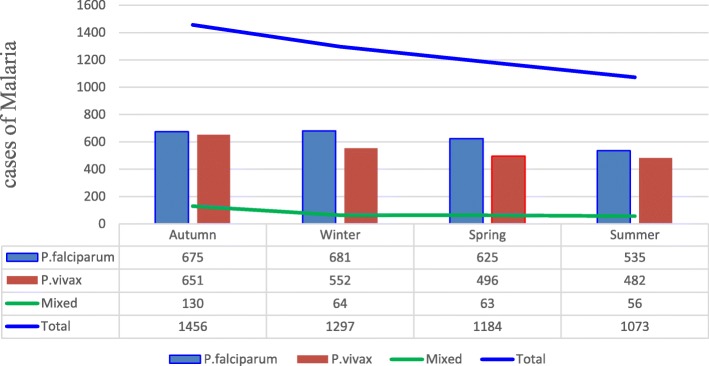
Distribution of plasmodium species with different seasons in Suhul General Hospital from 2012 to 2018

We analyzed the seasonal variation of malaria in the study area. Malaria cases have been reported in all months and seasons. The outcome of this study showed that the highest number of malaria cases was observed in autumn (September–November) and the lowest during the summer season (Fig. 3). At species level, the highest number of *P.vivax* and mixed infections were recorded in autumn while *P.falciparum* was high during winter season. On the other hand, minimum cases of *P. falciparum*, *P. vivax* and mixed infections were observed in summer season (Fig. 4).

## Discussion

In this seven-year retrospective study, we assessed distribution of malaria among individuals by socio-demographics, climate, and species of plasmodium parasites. The finding of this study indicated that the number of confirmed malaria cases were 5010 with total slide positivity rate (SPR) of 6.96%. It is comparable with other studies conducted in Ethiopia such as Kombolicha (7.52%) [15], Bahirdar (5%) [16], Arsi Negelle (11.4%) [17], and Northern Shoa (8.4%) [18]. On the other hand, the prevalence of the present study is much higher than study conducted Saudi Arabia (0.1%) [19]. Mean while, the current malaria prevalence is lower than the study conducted in Welega zone (20.07%) [20], Wereta town (32.6%) [21], Kola Diba (39.6%) [22], and Omo zone of southern Ethiopia (41.5%) [23]. These differences in malaria prevalence might be due to differences in climate, skill of the laboratory personnel, endemicity of the study areas, types of malaria intervention activities and malaria diagnostic techniques used. The rising up of the malaria case in other part of Ethiopia in the above also due to the malaria outbreaks or high malaria transmission setting.

The number of malaria cases in the study area showed year-to-year fluctuation from January 2012-to December 2018. An increase in malaria cases was observed from 2014 to2016. In contrary, there was a decrease in malaria cases from the year 2017 to2018. The reduction of malaria cases from 2017 to 2018 might correspond with increased awareness of the community towards the application of different insecticides and repellents (like buzz of), improving quality of indoor residual spray operation and minimizing environmental compliance. This is because, Ethiopia currently will plan to eliminate malaria by 2030 in collaboration with different stakeholders so that the community have strong willing and initiation of malaria prevention and control than the past decade years.

In this study, males were more affected by malaria than females. This is parallel with earlier studies carried out in eastern Wollega [20], southwest Ethiopia [24] and south central Ethiopia [25]. In contrast, lower malaria cases in males were reported from studies in Kenya [26] and Mozambique [27]. Due to the fact that in our study gold mining activities are mainly practiced. So, most of the time males were engaged themselves for such activities by staying outdoors during night and early evening to extract and keep their products when mosquito becomes active to find meal. Hence, have got higher chance of exposure to be infected by the anopheles malaria vector.

Although malaria cases were reported in all age groups, individuals aged fifteen years or older were more affected with the prevalence of 78.2%. This was in agreement with the study conducted in other parts of Ethiopia like Welega [20] and south central Ethiopia [25]. On the other hand, higher prevalence of malaria was reported among individuals less than 5 years of age [28, 29]. The reported lower prevalence of malaria in children under 14 years of age might be because of their less likely exposure to infected mosquito bite due to less likely to engage outdoor activities when compare with the older one.

In this study, malaria cases were reported in all of the four seasons in Ethiopia. This is because malaria transmission is not solely determined by season rather social, biological and economical factors such as mosquito control measures, population immunity, governmental policy and drug resistance have also contribution on malaria transmission and prevalence. The highest number of cases and detection rate of malaria were observed in autumn (September to November). This finding is in line with the study conducted in Debremarkos [30], Oromia [31], Wolaita zone [32] and northern Shoa [18]. This might be due to existence of favorable local conditions like stagnant water and micro-environmental or host spots following falling of significant rain followed by dry season which create conducive environment for rising of mosquito population, survival of the parasite in the mosquito, biting rate and transmission of malaria parasites.

## Conclusions

Malaria is still a major health dilemma in northwestern Tigrai, northern Ethiopia. *Plasmodium falciparum* and *Plasmodium vivax* were unmoving predominant parasite detected. The highest peak of malaria cases was observed during the autumn (September–November) seasons and males are more affected than females in the study area. Overall, trend of malaria over the years showed no significant reduction or increment. So, strong scaling up of the community should going on towards transmission, prevention and control activities of malaria in view of *Plasmodium falciparum* and *Plasmodium vivax.*

### Limitation of the study

The present study was restricted to the hospital recorded retrospective data on age and sex and was unable to relate to the current prevalence status of malaria in the study area. Few data were incomplete.

## Data Availability

To generate findings of this study, data was collected and analyzed based on the stated methods and materials. All the data were incorporated in the manuscript and no supplementary files accompanied the submission. The original data supporting this finding will be available at any time upon request.

## Abbreviations

FMOH: Federal ministry of health
HAD: Health development army
HEW: Health extension workers
HMIS: Health management information system
OPD: Outpatient department
SPSS: Statistical product and service solutions
WHO: World health organization
